# Effects of the COVID-19 pandemic on thoracic sympathectomies performed on Brazil’s Public Health System

**DOI:** 10.1590/1677-5449.202401192

**Published:** 2026-02-09

**Authors:** Carolina Carvalho Jansen Sorbello, Felipe Soares Oliveira Portela, Marcelo Fiorelli Alexandrino da Silva, Marcelo Passos Teivelis, Giulia de Payrebrune St Séve Marins Girardi, José Ribas Milanez de Campos, Nelson Wolosker

**Affiliations:** 1 Hospital Israelita Albert Einstein, São Paulo, SP, Brasil.; 2 Faculdade Israelita de Ciências da Saúde Albert Einstein – FICSAE, São Paulo, SP, Brasil.; 3 Universidade de São Paulo – USP, Faculdade de Medicina, São Paulo, SP, Brasil.

**Keywords:** big data, hyperhidrosis, thoracoscopy, sympathectomy, public health, COVID-19, big data, hiperidrose, toracoscopia, simpatectomia, saúde pública, covid-19

## Abstract

**Background:**

The COVID-19 pandemic affected health resources in Brazil, as priority was given to providing care to those infected. As a result, patients with other pathologies suffered delays in treatment, especially those waiting for elective surgical treatments, such as sympathectomy for hyperhidrosis.

**Objectives:**

To analyze the number of thoracic sympathectomies performed to treat hyperhidrosis on the Brazilian Unified Health System (SUS), their mortality, demographics, length of hospital stay, and associated costs in the periods before, during, and after the peak of the COVID-19 pandemic.

**Methods:**

This is a retrospective cross-sectional study that analyzed the number of video-assisted thoracic sympathectomies performed on the SUS in the 2 years prior to the pandemic (2018 and 2019), the 2 years of peak pandemic (2020 and 2021), and the 2 years after (2022 and 2023). Data on regional distribution, profile of patients, hospitalization, and associated mortality were also analyzed. Figures were extracted from the SUS Department of Informatics (DATASUS) database.

**Results:**

The number of sympathectomies performed in Brazil during 2020 and 2021 was 60% lower than the number recorded in 2018 and 2019, followed by a 30% smaller than expected increase in 2022 and 2023. Half of these procedures were conducted in the Southeast region of the country. The profile of patients was predominantly young women. Hospitalization time, costs, and mortality were stable over the years.

**Conclusions:**

The volume of sympathectomies for treatment of hyperhidrosis in the SUS was severely affected by the COVID-19 pandemic and remains in slow recovery. The regional distribution, demographic profile, hospitalization data, and mortality rate remained stable.

## Resumo

**Contexto:**

A pandemia de covid-19 afetou os recursos de saúde no Brasil, com a priorização do atendimento aos pacientes infectados. Como resultado, outras doenças sofreram atrasos no tratamento, especialmente os procedimentos cirúrgicos eletivos, como as simpatectomias para hiperidrose.

**Objetivos:**

Analisar o número de simpatectomias torácicas realizadas para o tratamento de hiperidrose no Sistema Único de Saúde (SUS), bem como a taxa de mortalidade, os dados demográficos, o tempo de internação e os custos associados nos períodos antes, durante e após o pico da pandemia de covid-19.

**Métodos:**

Trata-se de um estudo transversal retrospectivo, que analisou o número de simpatectomias torácicas videoassistidas realizadas no SUS nos 2 anos anteriores à pandemia (2018-2019), bem como nos 2 anos de pico (2020-2021) e nos 2 anos posteriores (2022-2023). Também foram analisados a distribuição regional, o perfil dos pacientes, os dados de internação e a mortalidade associada. Os dados foram extraídos do banco de dados do Departamento de Informática do SUS.

**Resultados:**

O número de simpatectomias realizadas em 2020-2021 foi 60% menor do que o registrado em 2018-2019, seguido de um aumento 30% menor do que o esperado em 2022-2023. Metade dos procedimentos ocorreu na região Sudeste. O perfil dos pacientes é de mulheres jovens. O tempo de internação, os custos e a mortalidade se mantiveram estáveis ao longo dos anos.

**Conclusões:**

O volume de simpatectomias para tratamento de hiperidrose no SUS foi extremamente impactado pela pandemia de covid-19 e ainda permanece em lenta recuperação. A distribuição regional, o perfil demográfico, os dados de internação e a mortalidade permaneceram estáveis.

## INTRODUCTION

Hyperhidrosis is a condition that seriously compromises the personal^1^ and professional life and mental well-being^2^ of individuals who experience it. Most people with this condition are teenagers or young adults who are at the peak of their productive lives.^3^ When the correct indications for surgical treatment with sympathectomy are present, risk is low^4^ and the procedure improves quality of life in over 90% of cases.^5^ Hyperhidrosis is the most common indication for thoracic sympathectomy,^6^ but upper limb ischemia, post-traumatic chest pain syndromes, and refractory Raynaud’s phenomenon may also constitute indications for sympathectomy in some rare cases.^7^

While the excessive sweating that characterizes hyperhidrosis does not result in lethal outcomes, it can cause considerable morbidity, triggering or exacerbating psychological disorders,^8^ causing dermatological problems such as dermatitis, and impairing the academic and professional development of those affected. The effects on quality of life can be even greater in childhood and adulthood, increasing rates of bullying and harming school performance. Some less invasive therapeutic options exist, such as botulinum toxin injection, especially for axillary hyperhidrosis, and oxybutynin^9^ for palmoplantar hyperhidrosis, but thoracic sympathectomy is the only definitive treatment.

In Brazil, approximately 75% of the population, which is around 170 million people, depend on the public system (SUS)^10^ to meet their health care needs. Data regarding public health in Brazil, including information about surgical procedures carried out by the SUS (Unified Health System), is stored and managed on a single portal belonging to the Unified Health System’s Department of Information and Informatics (DATASUS).^11^ The Digital Health and Information Secretariat manages this system, which stores data on health indicators, health care, epidemiological and morbidity information, information on the health care network, vital statistics, demographic and socio-economic information, financial information, and public health costs. The data is anonymized and available on the DATASUS online platform, which was the source of the data collected for this study.

Louzada et al.,^12^ conducted a nationwide cross-sectional study analyzing 12 years of data on thoracic sympathectomy in Brazil.^13^ They found that 13 201 endoscopic thoracic sympathectomies were performed in the country from 2008 to 2019, with a rate of 68.44 procedures per 10 million inhabitants per year. Silva et al.^14^ conducted another population-based cross-sectional study, showing that the number of sympathectomies decreased significantly over 11 years (P = 0.001), probably due to increased use of Oxybutynin as a drug treatment.^15^

In 2020 and 2021, Brazil became one of the global epicenters of the COVID-19 pandemic,^16^ with over 38 million cases and more than 700 000 deaths associated with the disease and countless complications, mainly respiratory, hematological and vascular, which are still under study and have caused great morbidity among those affected.^17^ Development of a vaccine in record time by mid-2020 marked a breakthrough in preventing the disease and controlling the pandemic, given that there were still no specific treatment guidelines in the literature. The rapid spread of the lethal virus forced the health system to reorganize itself to deal with respiratory syndrome cases^18^ and elective surgical treatments, such as sympathectomies, were unavoidably postponed. This caused a backlog of patients and further impacted these individuals’ quality of life.

Although some previous studies have evaluated the negative impact on elective surgeries during the peak of the COVID-19 pandemic, there is a gap in our understanding. No study has yet evaluated the trend in the number of sympathectomies after the end of the COVID-19 pandemic.^19^ This underscores the need for further research to fully comprehend the long-term effects of the pandemic on our health care system.

This study used DATASUS data to analyze the number of thoracic sympathectomies for the treatment of hyperhidrosis in the Unified Health System (SUS), their mortality, demographics, length of hospital stay, and associated costs in the periods before (2018 and 2019), during (2020 and 2021), and after (2022 and 2023) the peak of the COVID-19 pandemic.

## METHODS

This was a study for which we analyzed data obtained from DATASUS, a Ministry of Health platform that gathers information on hospital admissions financed by the SUS. The study was approved by the institution’s Research Ethics Committee. This platform is public access and is populated with anonymized data, so there was no need to employ informed consent forms.

### Period analyzed, data source, and data extraction

This retrospective study analyzed the available data on thoracic sympathectomies performed on Brazil’s public health network between 2018 and 2023. The data were obtained from DATASUS, a digital platform run by the Unified Health System that provides open access data on procedures carried out in public hospitals accredited by the system. Accreditation is obligatory for institutions to be eligible for government reimbursement.

All the data were collected from this platform using an automated extraction method programmed by the institution’s IT service in the Python language (v. 2.7.13; Beaverton, OR, USA), using the Windows 10 operating system. Field selection on the DATASUS platform and subsequent table adjustment were carried out using Selenium WebDriver (v. 3.1.8; Selenium HQ) and Pandas (v. 2.7.13; Lambda Foundry, Inc. and PyData Development Team, NY, USA).

### Selection of thoracic sympathectomy procedures on the platform

To analyze surgical procedures, we used the specific SIGTAP / SUS (Management System for Procedures, Medicines, Orthoses, Prostheses and Materials of the Unified Health System) code for VIDEO-ASSISTED THORACIC SYMPATHECTOMY (04.03.05.014-6). Morbidity and mortality data were extracted from the DATASUS / Ministry of Health platform.

Details of the website where the searches were carried out: Fundação Oswaldo Cruz^20^.

Filters: Row: “procedure performed”; “region of establishment”; “sex”; “age group”; “days of stay”.

Column: “year of processing”.

Measures: ''number of AIH''; ''number of deaths''; ''total value''; ''hospitalizations with ICU use''.

Period studied: January 2018 to December 2023*.*

For the procedure selected, demographic data (distribution of sympathectomies by region of the country, age group, and gender of patients), as well as mortality rate associated with the procedure, data on length of hospital and ICU stays, and reimbursement amounts passed on to institutions for the surgeries performed were collected.

The data were compiled in .cvs format and organized into tables using Microsoft Office Excel 2016 (Redmond, WA, USA). Data on the country’s population by age group were obtained from the website of the Brazilian Institute of Geography and Statistics (IBGE). Standardized rate calculations were based on the population exclusively dependent on Unified Health System (SUS) services, published periodically by the National Health Agency (ANS), based on data released in December of each year.

### Ethics committee approval and statistical analysis

This research was approved by the institution’s Research Ethics Committee under CAAAE number 35826320.2.0000.0071 and consubstantiated opinion number 4.321.508. The data were obtained anonymously through the DATASUS platform, so consent forms were unnecessary. The project was approved by the institution’s ethics committee.

For this study, we initially analyzed the number of video-assisted thoracic sympathectomies performed in the SUS from 2018 to 2023, along with the number of sympathectomies divided by macro-region, age, gender and race over the same period. The length of stay, costs, and clinical-surgical outcomes of patients undergoing sympathectomy in these 6 years were also analyzed. Additionally, the volume of sympathectomies was analyzed to compare 3 periods: before the COVID-19 pandemic (2018-2019), during the height of the pandemic (2020-2021), and after the COVID-19 outbreak (2022-2023).

The statistical analysis of the differences over the years and the comparisons between regions and sexes were assessed using Poisson regression models, with results presented as percentage differences and 95% confidence intervals (95% CI). The analyses were carried out using R version 4.4.1, with a 5% significance level.

## RESULTS

The following were analyzed sequentially: the number of thoracic sympathectomies performed to treat hyperhidrosis on the Unified Health System (SUS), their mortality, demographics, length of hospital stay, and associated costs in the pre-pandemic years (2018 and 2019), compared to the periods during the pandemic (2020 and 2021) and after (2022- 2023) the COVID-19 outbreak affected Brazil’s public health system. Analysis of the data showed that the number of thoracic sympathectomies performed on Brazil’s public health system fell by 60% when compared to the pre-pandemic period, although mortality related to these procedures remained stable and low in all periods. In addition, the majority of patients undergoing sympathectomy, 65% of the entire sample, were female and in the adult age group (between 15 and 59 years old), with no changes during the pandemic. Finally, the analysis showed that the length of hospital stay did not change significantly, with a slight increase in the Southeast region. The reimbursement sums passed on to the health system also remained stable at an average of R$1,115.27 per procedure, with no significant differences. A detailed evaluation of the results follows below.

**Figure 1:**
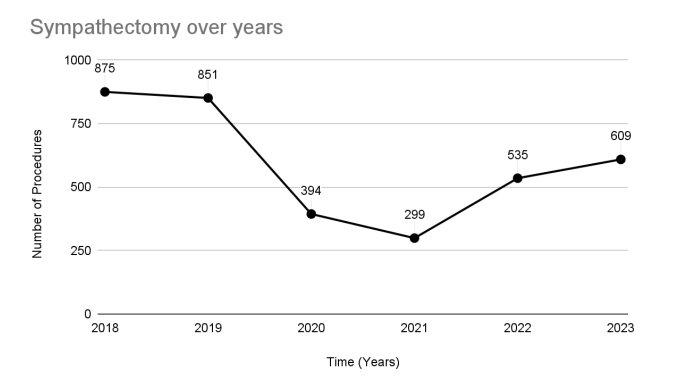
Temporal trend in the number of sympathectomies in Brazil over 3 periods: pre-pandemic (2018 and 2019), during the pandemic (2020 and 2021), and post-pandemic (2022-2023).

A temporal analysis of the number of sympathectomies shows that the number of procedures decreased compared to the pre-pandemic years, with a drop of approximately 60% in the number of thoracic sympathectomies in 2020 and 2021 compared to 2018 and 2019. After the pandemic, with full resumption of elective surgeries, there was a trend towards an increase in sympathectomies, but rates remained lower than pre-pandemic levels. In 2022 and 2023 there was an increase in the number of procedures, but this is still lower than in the initial years, with a drop of approximately 34% compared to pre-pandemic levels. When we evaluate the number of procedures over the years, we estimate an average reduction of 10.9% per year (95% CI 9.1% to 12.6%, p-value <0.001). Figure 1 illustrates the temporal analysis of the number of sympathectomies over the years.

**Figure 2:**
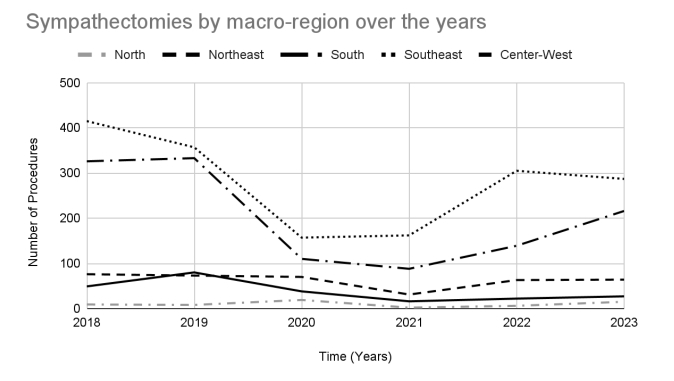
Temporal trend in the number of sympathectomies in Brazil, divided by macro-region, in 3 periods: pre-pandemic (2018 and 2019), during the pandemic (2020 and 2021), and post-pandemic (2022-2023) (North, Northeast, South, Southeast, and Midwest).

For an analysis that included socioeconomic aspects and social inequalities, the numbers of sympathectomies were analyzed by the macro-regions of Brazil. Figure 2 presents an analysis of the number of sympathectomies performed per macro-region. When we evaluate the regions, the highest number of procedures was in the Southeast region. Compared to this region, the numbers are 28.0% lower in the South (95% CI: 22.5% to 33.1%), 77.6% lower in the Northeast (95% CI: 75.0% to 80.0%), 86.2% lower in the Midwest (95% CI: 84.2% to 88.0%), and 96.5% lower in the North (95% CI: 95.5% to 97.3%). It is important to note that the pandemic (2020-2021) had a negative impact on surgery volumes and this trend was observed in all regions except the North.

Mortality related to thoracic sympathectomy was low, with no deaths recorded in some regions throughout the analysis period, in 2018 only 1 death was recorded, in the Southeast region, in 2020 1 death in the Midwest region and 1 death in the Southeast region were recorded, totaling 3 deaths associated with thoracic sympathectomy in the period from 2018 to 2023. The South, North, and Northeast regions recorded no deaths related to sympathectomy at any point during the study period.

**Figure 3:**
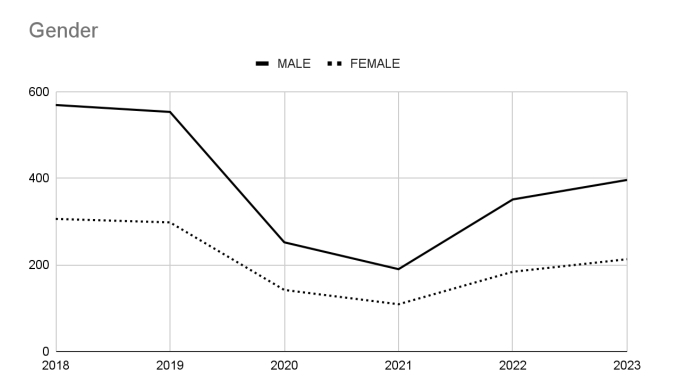
Temporal analysis of the gender profile of patients undergoing sympathectomy from 2018 to 2023.

**Figure 4:**
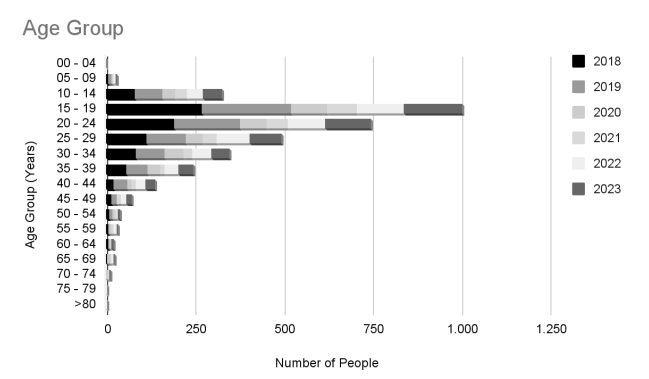
Temporal analysis of the age of patients undergoing sympathectomy from 2018 to 2023.

The description of the biological sex and age groups of the patients who underwent sympathectomies during the periods observed are shown in Figures 3 and 4, respectively. We conclude that the number of procedures in women was 84.6% higher than in men (95% CI: 72.4% to 97.8%). The majority of the patients who underwent surgery were between 15 and 59 years old.

**Figure 5:**
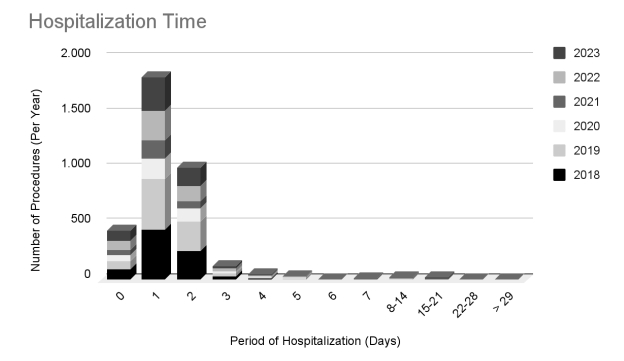
Temporal analysis of length of hospital stay related to sympathectomy, from 2018 to 2023. Horizontal: days in hospital. Vertical: number of patients.

Clinical and surgical aspects related to sympathectomy, such as the length of hospital stay and the amounts transferred to the health system per procedure, are illustrated in Table 1 and Figure 5. This analysis shows that the length of hospital stay was less than 3 days in 90% of cases throughout the analyzed period. Additionally, the cost per surgery remained stable over the years, remaining below R$1,500.00.

**Table 1:** Temporal analysis of costs per sympathectomy procedure, by macro-region of Brazil, from 2018 to 2023.

**Region / Year**	**2018**	**2019**	**2020**	**2021**	**2022**	**2023**
North	R$1,028.98	R$1,030.15	R$1,029.86	R$1,032.52	R$1,039.33	R$1,076.46
Northeast	R$1,067.28	R$1,061.55	R$1,133.65	R$1,190.95	R$1,107.85	R$1,134.70
South	R$1,031.01	R$1,050.14	R$1,102.88	R$1,033.21	R$1,136.58	R$1,058.39
Southeast	R$1,055.68	R$1,059.86	R$1,369.95	R$1,220.46	R$1,120.67	R$1,115.30
Midwest	R$1,126.61	R$1,104.86	R$1,192.07	R$1,311.27	R$1,068.63	R$1,144.75

During the peak of the pandemic, sympathectomy-related ICU use only increased in the Southeast. On the other hand, in the other regions, the number of patients admitted to ICUs remained stable. Moreover, there was no significant difference in the number of deaths over the six years, remaining low throughout (less than 1%).

## DISCUSSION

This nationwide epidemiological analysis of the number of video-assisted sympathectomies conducted on Brazil’s public health system in the periods before, during, and after the COVID-19 pandemic revealed a drop in the number of procedures with a lower-than-expected increase after the virus had been controlled. There was a predominance of surgeries in the Southeast region. There were no changes in the age or gender of the patients affected, in the length of hospital stay, or in associated costs despite a slight increase in ICU admissions in the Southeast region.

The COVID-19 pandemic caused significant changes to health services across the globe^21^ as resources were reassigned to care for patients affected by the coronavirus. Brazil is no exception to this, as emergency care was prioritized, with elective surgeries and minor medical care receiving less attention.^22^

During the COVID-19 pandemic, medical societies, including the American College of Surgeons, classified surgical procedures into different groups. They recommended suspending elective surgeries,^23^ especially those for diseases that have lower potential for morbidity and mortality, like sympathectomies for hyperhidrosis.^24^ As a result, "purely elective" surgeries were initially canceled in nearly all specialties, leading to the postponement of over 900,000 elective surgeries in Brazil in 2020 alone.^25^ Some of these surgeries had started to resume by the end of 2021.

Postponement of non-urgent medical procedures during the pandemic has had consequences for both patients and the healthcare system. Patients suffered prolonged delays, which affected their quality of life and productivity and, in some cases, culminated in progression of the disease. For the health system, the backlog of cases meant increased spending in the post-pandemic period to reduce the waiting list for surgeries.

Although the number of sympathectomies has slowly returned towards pre-pandemic levels, it is still significantly lower than the average in the pre-pandemic period. There are several possible reasons for this, including the economic crisis resulting from the pandemic, the need to prioritize more urgent procedures, loss of patients to follow-up, and limited access to health services. In addition, drug-based treatment for hyperhidrosis with oxybutynin may have contributed to a reduction in surgical treatment.^26^

The number of sympathectomies varies by region, with more developed regions recovering more quickly in the post-pandemic period than less developed regions. Thus, states in the South and Southeast regions of Brazil experienced a faster and more effective economic recovery in the post-pandemic period, facilitating reinvestment in elective surgeries. In these regions, the higher social and educational level, access to information and health services, and greater urban concentration may also have influenced the higher prevalence of sympathectomies.

The demographic profile of patients undergoing sympathectomies remained stable before, during, and after the pandemic. Peak incidence was among young patients aged from 15 to 30 years, with a majority of females.^27,28^ Young patients, who suffer from hyperhidrosis and have a lower surgical risk, suffer a significant social impact and, therefore, exhibit a greater demand for treatment.^29^

This study reaffirms the previously established facts regarding video-assisted thoracic sympathectomies. This procedure has low morbidity and mortality rates and requires only a short stay in hospital.^30^ Ideally, patients should be admitted the day prior to the procedure and discharged on the first day after the surgery, following a brief stay in the ward. This reduces unnecessary hospital occupancy and avoidable costs. In this context, sympathectomy is a low-cost surgery,^31^ with a cost of approximately 300 dollars per procedure, with little change in values during the pandemic despite the shortage of essential supplies.

Due to the COVID-19 outbreak, several types of surgery of varying complexity were suspended to prioritize treatment of patients with the potential to experience severe morbidity. This resulted in significant delays for many procedures. As we return to normal, surgeries for more severe diseases such as cancer, transplants, and heart surgery, are prioritized over procedures like sympathectomies. Despite its considerable impact on patients’ quality of life,^32^ hyperhidrosis is not typically life-threatening^33^ and will likely take longer to return to pre-pandemic levels than other elective procedures.

In order to minimize the delay in elective procedures, it is important to resume outpatient follow-up, proactively attempting to locate patients if necessary, and to reorganize the surgery waiting list according to the severity of the pathology to be treated electively. ‘Surgical task forces’ can be a valuable strategy in this context.

It is important to point out some limitations of this study. Like any population-based study, there is a risk of information bias since the data are reliant on the records of healthcare professionals, which can result in filling in errors, especially of procedure codes, underestimating the numbers. In addition, it is not possible to define the exact size of the sample, since the entire population living in Brazil has access to the SUS but does not necessarily use its services, although it is known that more than 140 million inhabitants depend exclusively on the public health care service. Finally, the period of the COVID-19 pandemic represented a time of psychosocial instability, especially for the healthcare professionals responsible for recording information, which may also have influenced the data, albeit in an insignificant way when compared to the size of the sample studied. Even so, further epidemiological studies are of the utmost importance to confirm the findings.

## CONCLUSION

The COVID-19 pandemic has had a severe impact on the number of video-assisted thoracic sympathectomies being performed. Although there has been an upward trend in the numbers of this kind of surgery, it is unlikely that we will see a return to pre-pandemic levels anytime soon. However, the pandemic has not significantly affected the demographic profile, costs per procedure, length of hospital stay, or mortality associated with sympathectomy.

## References

[1] Estevan FA, Wolosker MB, Wolosker N, Puech-Leão P (2017). Epidemiologic analysis of prevalence of the hyperhidrosis. An Bras Dermatol.

[2] Bahar R, Zhou P, Liu Y (2016). The prevalence of anxiety and depression in patients with or without hyperhidrosis (HH). J Am Acad Dermatol.

[3] Wolosker N, Krutman M, Kauffman P, Paula RP, Campos JRM, Puech-Leão P (2013). Effectiveness of oxybutynin for treatment of hyperhidrosis in overweight and obese patients. Rev Assoc Med Bras.

[4] Milanez de Campos JR, Kauffman P, Gomes O, Wolosker N (2016). Video-assisted thoracic sympathectomy for hyperhidrosis. Thorac Surg Clin.

[5] Wei Y, Xu ZD, Li H (2020). Quality of life after thoracic sympathectomy for palmar hyperhidrosis: a meta-analysis. Gen Thorac Cardiovasc Surg.

[6] Loureiro M, Lemos  AN, Salvalaggio PRO, Alwazzan M (2020). Minilaparoscopic lumbar sympathectomy with 3 mm instruments for plantar hyperhidrosis. J Vasc Bras.

[7] Kauffman P, Ribas Milanez de Campos J, Wolosker N (2003). Thoracoscopic cervicothoracic sympathectomy: an eight-year experience. J Vasc Bras.

[8] Loureiro MP, Novais PM, Coelho RM, Paulin JAN (2024). Sexual effects and longterm outcomes of endoscopic lumbar sympathectomy for plantar hyperhidrosis in men: a cross-sectional study. J Vasc Bras.

[9] Wolosker N, Faustino CB, Silva MFA, Campos JRM, Kauffman P (2020). Current treatment options for craniofacial hyperhidrosis. J Vasc Bras.

[10] Brasil (2024). Sistema Único de Saúde – SUS.

[11] Brasil (2024). Departamento de Informática do SUS (DATASUS).

[12] Louzada ACS, Silva MFA, Portugal MFC (2023). Nationwide cross-sectional analysis of endoscopic thoracic sympathectomy to treat hyperhidrosis over 12 years in Brazil: epidemiology, costs, and mortality. Ann Surg.

[13] Tetteh HA, Groth SS, Kast T (2009). Primary palmoplantar hyperhidrosis and thoracoscopic sympathectomy: a new objective assessment method. Ann Thorac Surg.

[14] Silva MFA, Louzada ACS, Teivelis MP (2022). Population-based analysis of the epidemiology of the surgical correction of hyperhidrosis in 1,216 patients over 11 years: a cross-sectional study. Sao Paulo Med J.

[15] Wolosker N, Campos JR, Kauffman P, Yazbek G, Neves S, Puech-Leao P (2013). Use of oxybutynin for treating plantar hyperhidrosis. Int J Dermatol.

[16] Steinman M, de Sousa JHB, Tustumi F, Wolosker N (2021). The burden of the pandemic on the non-SARS-CoV-2 emergencies: a multicenter study. Am J Emerg Med.

[17] Rizzo LV, Wolosker N (2020). Brazil’s COVID-19 response. Lancet.

[18] Dal Poz MR, Levcovitz E, Bahia L (2021). Brazil’s fight against COVID-19. Am J Public Health.

[19] Alexandrino da Silva MF, Oliveira Portela FS, Sposato Louzada AC, Teivelis MP, Amaro E, Wolosker N (2024). National cross-sectional epidemiological analysis of the impact of pandemic COVID-19 on vascular procedures in public health system: 521,069 procedures over 4 years. Ann Vasc Surg.

[20] Fundação Oswaldo Cruz (2024). Autorizações de Internação Hospitalar aprovadas.

[21] Wang H, Paulson KR, Pease SA (2022). Estimating excess mortality due to the COVID-19 pandemic: a systematic analysis of COVID-19-related mortality, 2020-21. Lancet.

[22] Oliveira WK, Cavalcanti LPG, Croda J (2022). Coronavirus disease COVID-19 pandemic and the Declaration of Public Health Emergency in Brazil: administrative and epidemiological aspects. Rev Soc Bras Med Trop.

[23] Cunha MJS, Silva MFA, Souza KP (2022). Effects of COVID-19 outbreak in image-guided biopsies in brazil: an epidemiological study over 13 years and 2 million biopsies. Cardiovasc Intervent Radiol.

[24] Wolosker N, Teivelis MP, Krutman M (2014). Long-term results of the use of oxybutynin for the treatment of axillary hyperhidrosis. Ann Vasc Surg.

[25] Rosin D (2022). COVID-19 effect on surgical care. Isr Med Assoc J.

[26] Wolosker N, Teivelis MP, Krutman M (2015). Long-term efficacy of oxybutynin for palmar and plantar hyperhidrosis in children younger than 14 Years. Pediatr Dermatol.

[27] Wolosker N, Munia MA, Kauffman P, Campos JR, Yazbek G, Puech-Leão P (2010). Is gender a predictive factor for satisfaction among patients undergoing sympathectomy to treat palmar hyperhidrosis?. Clinics.

[28] Wolosker N, Krutman M, Campdell TPDA, Kauffman P, de Campos JRM, Puech-Leão P (2012). Oxybutynin treatment for hyperhidrosis: a comparative analysis between genders. Einstein.

[29] McConaghy JR, Fosselman D (2018). Hyperhidrosis: management options. Am Fam Physician.

[30] Milanez de Campos JR, Kauffman P, Wolosker N (2006). Axillary hyperhidrosis: T3/T4 versus T4 thoracic sympathectomy in a series of 276 cases. J Laparoendosc Adv Surg Tech A.

[31] Lima SO, Machado J, Fontes LM, Figueiredo MBGA, Santos JM, Santana VR (2023). Evaluation of quality of life (QOL) of young patients with primary hyperhidrosis (PH) before and after endoscopic thoracic sympathectomy (ETS). J Am Acad Dermatol.

[32] Parashar K, Adlam T, Potts G (2023). The impact of hyperhidrosis on quality of life: a review of the literature. Am J Clin Dermatol.

[33] Wolosker N, Yazbek G, de Campos JR (2010). Quality of life before surgery is a predictive factor for satisfaction among patients undergoing sympathectomy to treat hyperhidrosis. J Vasc Surg.

